# Enhancing Soil Resilience: Bacterial Alginate Hydrogel vs. Algal Alginate in Mitigating Agricultural Challenges

**DOI:** 10.3390/gels9120988

**Published:** 2023-12-17

**Authors:** Flavia Dorochesi, Cesar Barrientos-Sanhueza, Álvaro Díaz-Barrera, Italo F. Cuneo

**Affiliations:** 1 Facultad de Ciencias Agronómicas y de los Alimentos, Pontificia Universidad Católica de Valparaíso, Valparaíso 2340025, Chile; fl.dorochesi@gmail.com (F.D.); c.barrientos.sanhueza@gmail.com (C.B.-S.); 2 Escuela de Ingeniería Bioquímica, Pontificia Universidad Católica de Valparaíso, Valparaíso 2340025, Chile; alvaro.diaz@pucv.cl

**Keywords:** bacterial alginate hydrogel, algal alginate hydrogel, soil hydromechanics, soil conditioner, climate change

## Abstract

Erosion and tillage changes negatively the soil physical structure, which directly impacts agricultural systems and consequently food security. To mitigate these adverse modifications, different polymeric materials from synthetic and natural sources, have been used as soil conditioners to improve the hydro-mechanical behavior of affected soils. One of the most interesting and used natural polymers is the alginate hydrogel. Although commercially available alginate hydrogels are primarily sourced from algal, they can also be sourced from bacteria. The gelation capacity of these hydrogels is determined by their molecular properties, which, in turn, are influenced by the production conditions. Bacterial alginate hydrogel production offers the advantage of precise control over environmental conditions during cultivation and extraction, thereby maintaining and enhancing their molecular properties. This, in turn, results in higher molecular weight and improved gelation capacity. In this study, we compared the effects of bacterial alginate (BH) and algal alginate (AH) hydrogels over the mechanical, hydraulic, and structural behavior of coarse quartz sand as a model soil. Mechanically, it was observed that the treatment with the lowest concentration of bacteria alginate hydrogel (BH1) reached higher values of yield strength, Young’s modulus (E), shear modulus (G) and strain energy (U) than those treatments with algal alginate hydrogel (AH). Furthermore, the increase in the aggregate stability could be associated with the improvement of mechanical parameters. On the other hand, a greater water retention capacity was observed in the BH treatments, as well as a greater decrease in hydraulic conductivity with respect to the AH and control treatments. All these changes could be explained by the formation of bridge-like structures between the sand particles and the hydrogel, and this alteration may result in a shift in the mechanical and wettability characteristics of the treated soils. Finally, our findings emphasize the superior impact of bacterial alginate hydrogel on enhancing the mechanical and hydraulic properties of coarse quartz sand compared to traditional algal alginate. Besides, the use of bacterial alginate hydrogel could be useful to counteract erosion and water scarcity scenarios in agricultural systems.

## 1. Introduction

Severe climate conditions and soil erosion, driven by climate change and human activities, pose a significant threat to the sustainability of agricultural soils and global crop production [1,2]. Addressing the need for implementing agricultural systems resilient to extreme drought and erosion is essential [3]. Soil resilience primarily hinges on its structure, defined as the organization of soil particles that gives rise to an intricate porous material comprising soil aggregates [3,4]. Soil properties and processes, such as infiltration, water holding capacity, drainage, gas exchange, organic matter content and root penetration into soil are controlled by the spatial structure of macro- and micropores [4,5,6]. In addition, soils constitute the habitat of a great variety of organisms, which consequently drives their diversity and regulates their activity [4,5,7]. Hence, the physical integrity of soil is fundamentally governed by its structure.

In agricultural systems, tillage is often the action that causes the greatest impact on soil structure [2,8]. Globally, an estimated 75 billion tons of fertile soil are lost annually due to management practices in agricultural systems [1]. Also, in areas under extreme drought events due to climate change, soils are more prone to structural failure due to the unexpected climatic events (e.g., mechanical impact of rain, heavy wind, hail) [3,9]. Consequently, the tilled soil with inadequate water content will lead to several problems such as mechanical disturbance, higher levels of compaction, and pulverization, enhancing soil erosion processes [2,10,11]. In order to mitigate these adverse effects on agricultural soils, it is imperative to devise novel strategies aimed at preserving or rejuvenating soil structure and stability, thereby safeguarding their essential functions [10].

Hydrogels are three dimensional, hydrophilic, polymeric materials with the ability to absorb and release large amounts of water, and possess glue-like effects, mimicking some of the physical effects of natural polymers in the soil (e.g., root mucilage, bacterial exopolysaccharides) [6,12,13,14,15]. Hydrogels have found extensive use in agriculture as soil conditioners due to their ability to enhance various hydro-mechanical properties of soil. This includes increasing water retention capacity, improving soil permeability and infiltration rates, reducing the need for frequent irrigation, minimizing compaction risks, and mitigating erosion caused by various environmental stressors [12,16,17,18,19]. According to the origin source, hydrogels can be classified as natural polymers or synthetic polymers [16,17]. At present, the majority of commercially accessible hydrogels are constructed from acrylic acid and polyacrylamide, which are synthetic polymers. Nonetheless, these materials are petroleum-derived, making them challenging to degrade in the soil [18,19]. In some cases, their derivate-degradation products could be biologically toxic [18]. Consequently, in the last few years a great interest has been shown in developing biodegradable hydrogels such as alginate [12,20], chitosan [21,22], and dextran [12,23]. These eco-friendly hydrogels have been employed in a range of applications due to their cost-effectiveness, sustainable nature, and biodegradable attributes [19,24]. Alginate hydrogel stands out as one of the most frequently utilized natural polymers owing to its remarkable characteristics, including hydrophilicity, biocompatibility, and biodegradability [12,20]. Alginate is mainly obtained from marine brown algae (Phaeophyceae), being one of their major constituents of algal cell walls [18,25]. Although, all the alginate commercially available is produced from the extraction of brown algae [26,27], it can also be synthesized by using bacteria from *Pseudomonas* and *Azotobacter* genera [28,29,30,31]. Alginates are linear polysaccharide that consists of (1-4)-linked β-D-mannuronate (M) and α-L-guluronate (G) residues [18,20]. The properties of alginate in a solution are contingent upon its monomer chemical composition (G/M ratio), the molecular weight (MW) of the resulting polymer, and any alterations or modifications made to the alginate (acetylation degree) [32,33,34,35,36]. The production of bacterial alginate can be developed using the bacterium *Azotobacter vinelandii* in bioreactors [31,32,33]. Controlled conditions of bacterial alginate production in bioreactors maintains its properties, unlike algal alginate (i.e., sodium alginate) whose composition and molecular weight, and consequently its efficiency, will depend on environmental conditions, which limits its use in industry [29,33,34]. Furthermore, different strategies have been developed to improve the bacterial alginate properties (i.e., degree of acetylation and molecular weight) under bioreactor conditions, to improve its gelation capacity [29,31,32,34,37]. In conclusion, the aforementioned properties render bacterial alginate a compelling soil conditioner suitable for integration into agricultural systems. Previous research has examined the impact of bacterial hydrogel on the hydro-mechanical characteristics of coarse-quartz sand [38,39]. Incorporating bacterial alginate hydrogel significantly enhanced the hydraulic conductivity, mechanical strength, and soil aggregate stability in the coarse-quartz sand [39]. Nonetheless, differences in the influence of bacterial alginate versus algal alginate on the physical properties of soils has not been definitively established thus far.

In here, we hypothesized that the addition of bacterial alginate hydrogel to the coarse quartz sand (i.e., model soil media) enhance the physical, mechanical, and hydraulic properties compared to algal alginate hydrogel. In order to evaluate our hypothesis, we conducted a comprehensive assessment involving several parameters. We measured pH values and electrical conductivity to characterize the chemical composition of various hydrogels. Unconfined uniaxial compression tests and aggregate stability measurements were carried out to assess the influence of these hydrogels on the mechanical properties of the soil. Furthermore, falling head permeability tests and water holding capacity measurements were employed to investigate their impact on fluid dynamics. Lastly, we utilized bright field microscopy in conjunction with contact angle measurements to analyze how the structural properties of these materials could potentially modify their wettability. The overarching aim of this study is to offer insights into identifying the most suitable alginate hydrogel for application in agricultural systems facing challenges due to climate change.

## 2. Results

### 2.1. pH and Electrical Conductivity

To evaluate the effects of bacterial (BH) and algal alginate (AH) hydrogels on coarse quartz sand, the pH and electrical conductivity were analyzed (Table 1; Figure S1). Three concentrations of each alginate hydrogel 0.05, 0.10 and 0.15% (*w*/*w*) were studied (i.e., AH1, AH2 and AH3, respectively). Significant differences were found in the pH and electrical conductivity measurements among treatments (*p* = 2.20 × 10^−16^ and 2.20 × 10^−16^, respectively). Bacterial alginate hydrogel (BH1, BH2 and BH3) presented a pH value 11% and 10% higher than the distilled water (W) and control treatment (C; Table 1).

The pH value remained unaffected by the concentration of bacterial alginate in the soil (Figure S1). In contrast, the AH1 and AH2 treatments exhibited significant disparities compared to AH3. Specifically, AH1 and AH2 displayed pH values approximately 5% and 6% lower than the control treatments, as indicated in Table 1. Conversely, the AH3 treatment exhibited a pH value that was approximately 3% and 4% lower than the W and C treatments (Figure S1). The electrical conductivity values of both alginate hydrogels presented statistical differences with control (i.e., W and C) treatments. Algal alginate hydrogels demonstrated 8.80 and 6.62 times higher electrical conductivity when compared to the W and C treatments. The concentration of alginate hydrogel did not exert any influence on the electrical conductivity of the samples. The treatments involving bacterial alginate hydrogels exhibited the highest electrical conductivity, with the increase being proportional to the concentrations of bacterial alginate. Specifically, BH1, BH2, and BH3 treatments recorded values that were 8.8, 13.6, and 18.1 times greater in comparison to the W and C treatments, respectively.

### 2.2. Unconfined Uniaxial Compression Test

**Figure 1:**
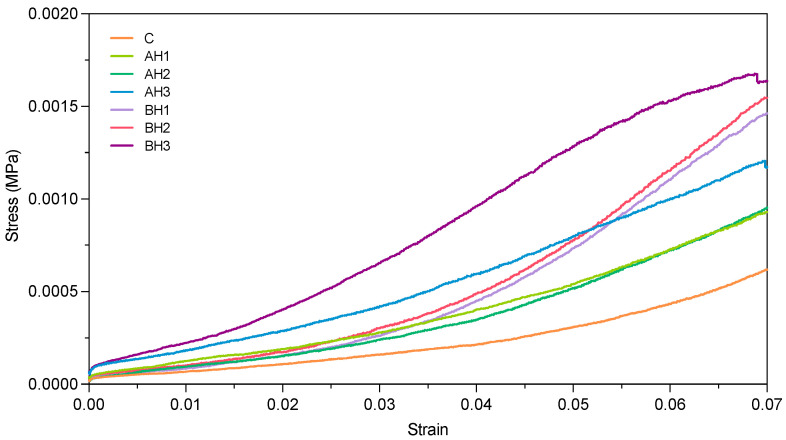
Representative stress-strain behavior showing the unconfined uniaxial compression test (UUCT) of control, bacterial alginate hydrogel (BH) and algal alginate hydrogel (AH) treatments (*n* = 5). Figure shows three alginates concentration levels 0.05%, 0.10% and 0.15% *w*/*w* (i.e., BH1, BH2, BH3, respectively). The stress-strain behavior of the samples was measured until the yield stress point was reached.

We used unconfined uniaxial compression test to quantify the mechanical behaviors of the different treatments. C, BH and AH treatments stress-strain relationship were measured (Figure 1). Significant differences were found in the stress behavior among treatments (*p* = 2.01 × 10^−16^).

Bacterial alginate hydrogel (BH1, BH2 and BH3) showed the highest yield stress compared to algal alginate hydrogel (AH1, AH2, and AH3) and control treatments (C), presenting statistical differences (Table 2; Figure S2). In the BH1 treatment (0.05% *w*/*w* alginate), the yield stress was 2.31 times higher than in the C treatment, and 1.58, 1.53, and 1.23 times higher than in the AH1, AH2, and AH3 treatments, respectively. In contrast, BH3 (0.15% *w*/*w* alginate) exhibited a yield stress 2.59 times greater than the C treatment and 1.76, 1.73, and 1.37 times higher than the AH1, AH2, and AH3 treatments, respectively. The algal alginate hydrogel treatments exhibited notable distinctions when compared to the control treatment, as indicated in Table 2 and Figure 1. Specifically, AH1, AH2, and AH3 demonstrated a 1.47, 1.51, and 1.89-fold increase in yield stress compared to the control treatment, respectively. 

The strain values for the treatments displayed a similar pattern, as detailed in Table 2. Furthermore, the samples’ elastic response to uniaxial compression, expressed in terms of the Young’s modulus (indicative of material stiffness or elastic behavior) [40], revealed statistically significant differences among the treatments (*p* = 2.2 × 10^−16^). Bacterial alginate hydrogel treatment showed the highest values compared with control and algal alginate hydrogel treatments (Table 2; Figure S2). The lowest concentration of bacterial alginate (BH1) showed 2.31, 1.58, 1.53 and 1.22-fold greater than the C, AH1, AH2 and AH3 treatments (i.e., lower values), respectively (Table 2). AH3 treatment presented significant differences with AH1 and AH2. Despite the above, all algal alginate treatments (AH1, AH2 y AH3) presented significant differences with the control treatment reaching 1.47, 1.51 and 1.90-fold greater to control treatment (Table 2; Figure S2). The shear modulus (G) measures the ratio of stress to strain under shear forces. Significant differences were found in shear modulus (G) among treatments (*p* = 2.2 × 10^−16^). The treatments presented a shear modulus (G) behavior as that reported in Young’s modulus (Table 2; Figure S2). The strain energy (U) allows us to classify and identify the elastic resilience among treatments displaying statistical differences (*p* = 2.0 × 10^−16^). Bacterial alginate hydrogel treatment did not present statistical differences and reached the highest values compared with control and algal alginate hydrogel treatments (Figure S2; Table 2). BH3 showed the most elastic resilience, reaching 1.36-fold compared with AH3 (Table 2). Regarding to the algal alginate hydrogel treatments, AH3 presented significant differences with AH1 and AH2, being 1.25 and 1.28-fold higher, respectively (Figure S2; Table 2).

### 2.3. Aggregate Stability Test

**Figure 2:**
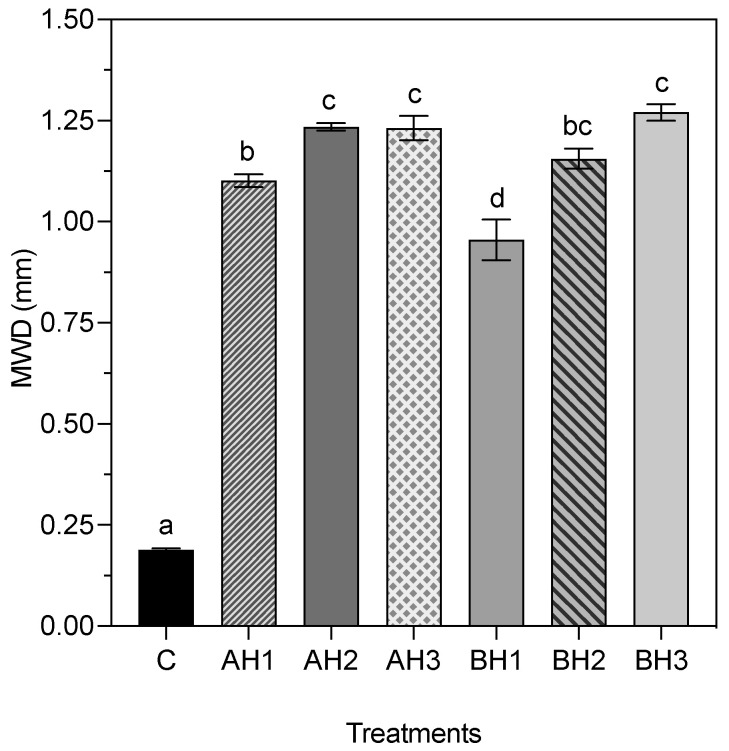
Stability of aggregates of the control and alginate hydrogels treatments. Mean weight diameter (MWD) was obtained using Equation (2). Data are means ± SE (*n* = 5). Mean followed by different letters are significantly different by Tukey test. (*p* < 0.05).

The mechanical breakdown of soil aggregates method was used to determine the aggregate stability between control and alginate hydrogel treatments. The treatments presented significant differences in aggregate stability (*p* = 2.10 × 10^−16^). BH and AH treatments exhibited significant disparities when compared to the control treatment, as depicted in Figure 2. Specifically, BH1 (0.96 ± 0.05 mm SE), BH2 (1.16 ± 0.03 mm SE), and BH3 (1.27 ± 0.02 mm SE) treatments achieved increases of 5.06, 6.12, and 6.72-fold, respectively, in comparison to the C treatment (Figure 2). A direct correlation was observed between aggregate stability and bacterial alginate concentration within the BH treatments. Furthermore, AH1 (1.10 ± 0.02 mm SE), AH2 (1.24 ± 0.01 mm SE), and AH3 (1.23 ± 0.03 mm SE) treatments recorded increases of 5.83, 6.54, and 6.52-fold, respectively, when compared to the C treatment (Figure 2). BH2, BH3, AH2, and AH3 treatments demonstrated the highest aggregate stability values, with no statistically significant differences among them. Notably, BH1 treatment displayed the lowest aggregate stability value among the alginate hydrogel treatments and exhibited significant distinctions when compared to all other treatments (Figure 2).

### 2.4. Soil Water Retention Capacity

**Figure 3:**
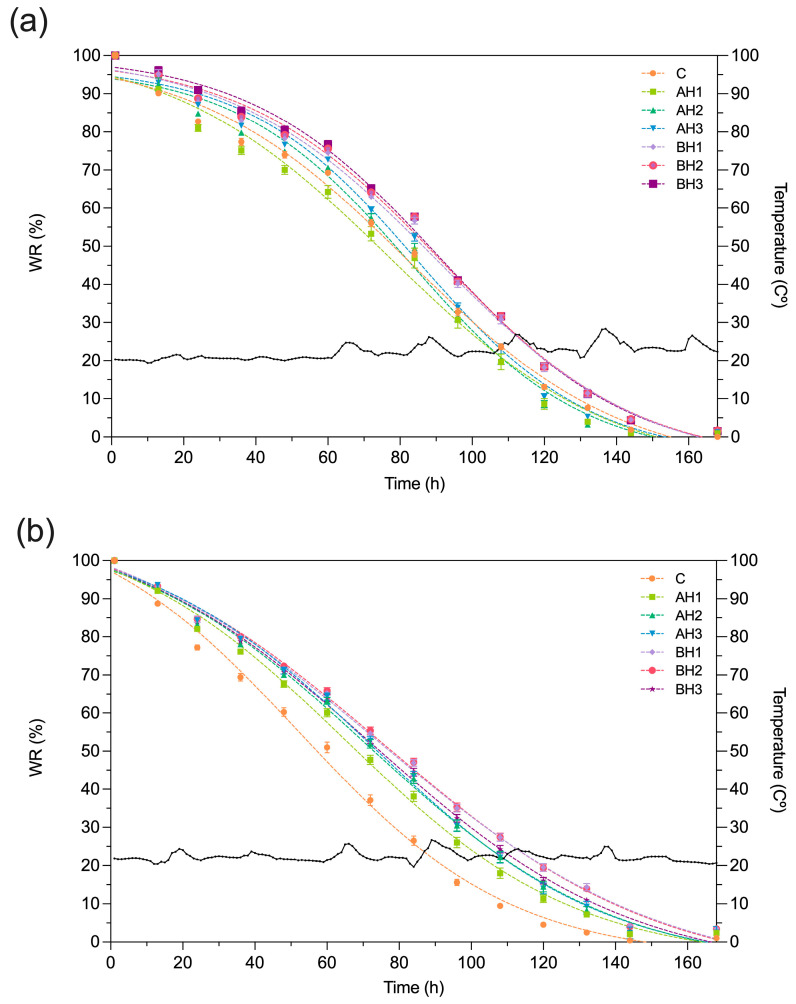
Effect of alginate concentration on water retention (WR) behavior. WR capacity of the treatments during the (**a**) first and (**b**) fourth swelling–drying cycles. The black line indicates the temperature during the test. Data are means ± SE (*n* = 5).

Assessing the water retention (WR) capabilities of C, BH and AH treatments is a critical step in gauging the potential of hydrogels as soil conditioners. This is particularly pertinent due to the capacity of hydrogels to enhance water retention, which is of great significance in scenarios marked by water scarcity [41,42]. To gain a deeper understanding of water retention capacity and the reusability of hydrogels within soil systems, multiple swelling-drying cycles were conducted. These cycles provide insights into the hydrogel’s ability to be effectively reused. Figure 3a illustrates the behavior of the treatments during the initial swelling-drying cycle. Significant differences were found among treatments at 24, 48, 72, 96, 120 and 144 h (*p* = 2.17 × 10^−11^, 8.77 × 10^−9^, 4.26 × 10^−8^, 5.96 × 10^−7^, 3.09 × 10^−9^, and 7.91 × 10^−9^, respectively). The BH treatments (BH1, BH2 and BH3) presented statistical differences with control treatment after 24 h (Figure S3a). BH3 reached the highest WR, showing 8.21%, 6.51%, 9.15%, 8.37% and 5.43% compared with control treatment at 24, 48, 72, 96 and 120 h. No statistical differences were observed between BH treatments (Figure S3a). AH1, AH2 and AH3 treatments showed a lower WR capacity compared to the BH treatments (Figure 3a). AH treatments did not display statistical differences with control treatment during the experiment, except at 24 and 48 h where AH1 presented a decreased WR of 1.8% and 3.8%, respectively. The treatment with the lowest concentration of bacterial alginate (0.05% *w*/*w*, BH1) showed 3.52%, 6.35%, 7.81% and 3.09% higher WR than the treatment with the highest concentration of algal alginate hydrogel (0.15% *w*/*w*; AH3) at 72, 96, 120 and 144 h, respectively. Different WR behaviors were found among treatments during the fourth swelling–drying cycle (Figure 3b). Significant differences were found at 24, 48, 72, 96, 120 and 144 h (*p* = 1.86 × 10^−9^, 2.19 × 10^−10^, 1.05 × 10^−11^, 2.36 × 10^−11^, 8.02 × 10^−10^, and 2.92 × 10^−7^, respectively). The BH treatments displayed a similar behavior as observed during the first swelling–drying cycle, where statistical differences with control treatment were obtained (Figure S3b). BH3 reached the highest WR values among the BH treatments with 6.77%, 10.55%, 15.86%, 16.81% and 11.51% more than control treatment at 24, 48, 72, 96 and 120 h, respectively (Figure S3b and Figure 3b). Contrary to the behavior observed during the first swelling–drying cycle, AH treatment presented significant differences with control treatment after 24 h (Figure S3b). The AH2 and AH3 treatments did not present statistical differences with the BH treatments until 96 h (Figure S3b). AH1 treatment displayed 4.90%, 7.40%, 10.60%, 10.46% and 6.79% higher WR than the control treatment at 24, 48, 72, 96 and 120 h, respectively (Figure S3b). The AH treatments presented changes in the WR capacity between the first and fourth swelling–drying cycles (Figure 3). The treatments showed greater WR capacity until 108 h in the first swelling–drying cycle (Figure S4).

The AH1 condition showed 4%, 12% and 18% higher WR at 48, 72 and 96 h during the first swelling–drying cycle (Figure S4a). AH2 and AH3 reached 9% and 14% higher WR at 48 and 72 h during the first swelling–drying cycle. Besides, at 96 h it was observed 3% and 11% higher WR, respectively (Figure S4c,e). BH1 and BH2 reached 9%, 16% and 15% higher WR (Figure S4b,d). BH3 presented 8%, 14%, 23%, and 27% higher WR at 24, 48, 72 and 96 h during first swelling–drying cycle, respectively (Figure S4f). After 120 h, a greater WR capacity was observed during the fourth swelling–drying cycle in the AH treatments (Figure 3 and Figure S4). AH1 and AH3 treatments reached 35% and 37% higher WR at 120 and 144 h during the fourth swelling–drying cycle. AH2 showed 33.1 and 78.9% higher WR at 120 and 144 h during the fourth swelling–drying cycle. The BH1 and BH2 treatments during the first and fourth cycles did not present changes at 120 h. However, at 144 h during the fourth swelling–drying cycle, 1.93% higher WR was observed. Finally, BH3 was the only condition that presented greater WR (15%) during the first swelling–drying cycle at 120 h.

### 2.5. Hydraulic Conductivity Test

**Figure 4:**
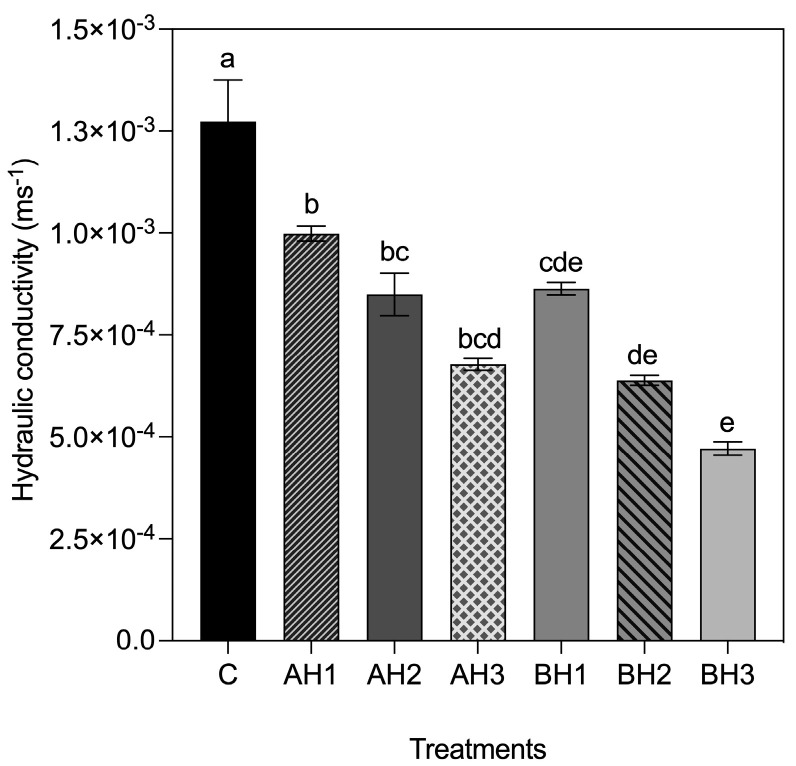
Hydraulic conductivity (k) of control treatment and bacterial and algal alginate hydrogels treatments was obtained with the falling head permeability test. Data are means ± SE (*n* = 5). Mean followed by different letters are significantly different by Tukey test. (*p* < 0.05).

To quantify the effects of the bacterial and algal alginate hydrogel concentration over the saturated hydraulic conductivity (*k*), we used the falling head permeability test. Significant differences were found in k values among treatments (*p* = 1.12 × 10^−10^). The BH1, BH2, BH3, AH1, AH2 and AH3 treatments showed a decrease saturated hydraulic conductivity of 32%, 50%, 63%, 22%, 33% and 44%, respectively, compared to the control treatment (Figure 4). The increase in concentration of alginate and the decrease in saturated hydraulic conductivity values was observed (Figure 4). The lowest k values were reached in BH3 (4.71 × 10^−4^ ± 1.68 × 10^−5^ ms^−1^ SE), BH2 (6.38 × 10^−4^ ± 1.23 × 10^−5^ ms^−1^ SE) and AH3 (6.77 × 10^−4^ ± 1.47 × 10^−5^ ms^−1^ SE) treatments and did not present statistical differences among them. BH3 treatment reached a k value of 1.83, 2.12 and 1.80-fold less than BH1 (8.63 × 10^−4^ ± 1.52 × 10^−5^ ms^−1^ SE), AH1 (9.98 × 10^−4^ ± 1.86 × 10^−5^ ms^−1^ SE) and AH2 (8.49 × 10^−4^ ± 5.21 × 10^−5^ ms^−1^ SE) treatments, respectively (Figure 4). While AH3 treatment showed a k value 1.27, 1.47 and 1.25-fold lower than BH1, AH1 and AH2, respectively (Figure 4).

### 2.6. Bright Field Light Microscopy Imaging

**Figure 5:**
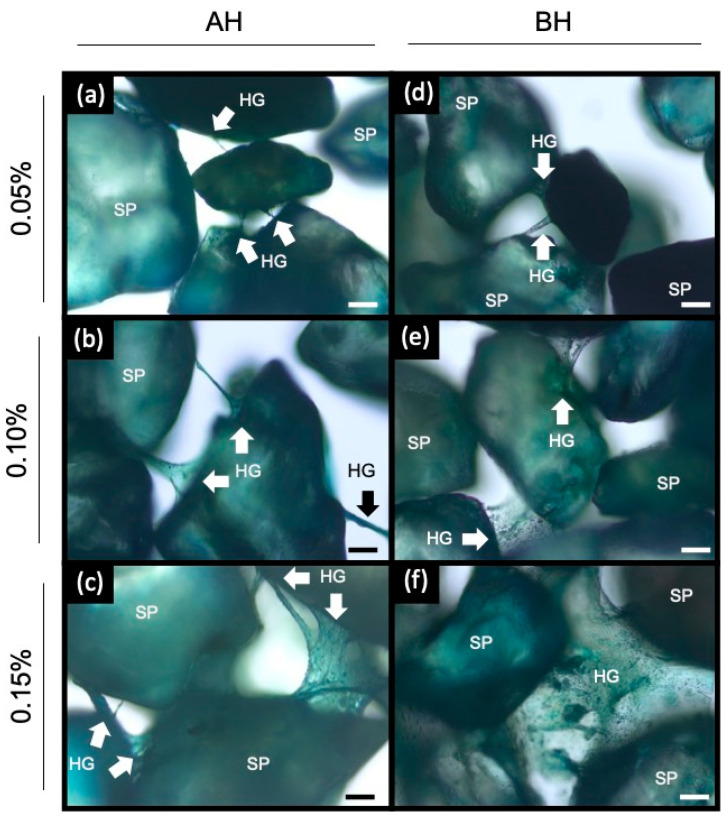
Bright field microscopy images of (**a**) AH1, (**b**) AH2, (**c**) AH3, (**d**) BH1, (**e**) BH2 and (**f**) BH3. A representative image of each treatment is shown. The treatments were covered with a blue-ink solution to generate contrast. Arrows indicate the alginate hydrogel bridges joining the sand particles. Bars = 500 μm; SP, sand particle; HG, alginate hydrogel.

To identify the structure of coarse quartz sand with different concentration of BH and AH treatments, bright field light microscopy imaging was used. We obtained 20 images per treatment and a representative image was selected for each treatment (Figure 5). The control treatment (sand without hydrogel) showed direct contact between the quartz sand particles, but without external connections of any kind [38,39]. Bacterial and algal alginate hydrogels treatments showed new links with the coarse sand particles (SP), forming a bridge-like structure between them (Figure 5). The addition of alginate hydrogel changes the structure of SP, showing a three-dimensional network. In the BH and AH higher concentration treatments, an increase in “SP-hydrogel-SP” interactions was observed (Figure 5). However, at the same alginate concentration, the BH treatments showed the formation of big sized bridges compared to the AH treatments (i.e., AH2 and BH2; Figure 5b,e).

### 2.7. Contact Angle Measurements

**Figure 6:**
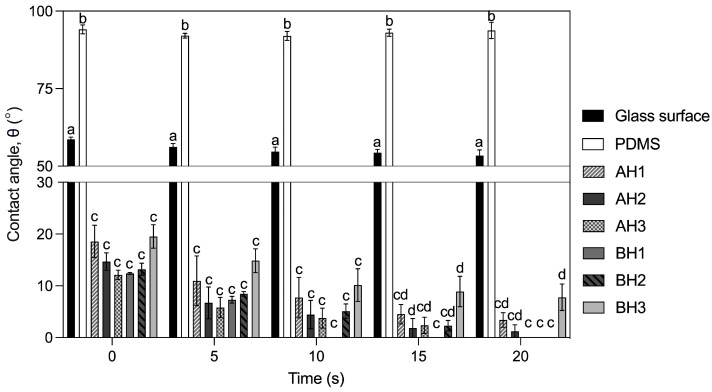
Measurement of contact angles (CA) for control treatment and alginate hydrogels treatment after 0, 5, 10, 15 and 20 s after the water drop was placed on the surface. Data are means ± SE (*n* = 6). Mean followed by different letters are significantly different by Tukey test. (*p* < 0.05).

The wettability characteristics of alginate hydrogels were assessed using the contact angle technique. This involved observing how water droplets behaved when placed on the surface of dried alginate hydrogels. To establish benchmarks for hydrophilicity and hydrophobicity, we utilized a glass slide surface and a glass slide coated with polydimethylsiloxane (PDMS), respectively. These choices were made based on their inherent physical interactions with water. Notably, our analysis revealed significant distinctions among the treatments (*p* = 2.20 × 10^−16^). At all times analyzed, the bacterial and algal alginate hydrogels presented a contact angle (<22°) lower than glass surface (53–56°) and PDMS (92–96°) materials, indicating that the hydrogels have a hydrophilic behavior (Figure 6). The contact angles measurements in algal alginate hydrogels treatments (AH1, AH2 and AH3), at the end of the test, showed a decrease of 77.90%, 89.43% and 99.73%, respectively. Algal alginate hydrogels did not present statistical differences (Figure 6). Bacterial alginate hydrogel treatments (BH1, BH2 and BH3) presented a decreased contact angle of 99.80%, 99.92% and 60.06%, respectively. At the end of the assay, AH3, BH1 and BH2 treatments reached a contact angle of 0° and presented significant differences with the BH3 treatment, condition that presented the highest value of contact angle (7.8°; Figure 6).

## 3. Discussion

This study aimed to assess the impact of bacterial alginate hydrogel (BH) in comparison with commercially available algal alginate hydrogel (AH) on the mechanical and hydraulic behavior of coarse quartz sand. In terms of mechanical properties, the BH3 treatment, which featured a higher concentration of bacterial alginate (0.15% *w*/*w*), exhibited notable increases in yield stress, Young’s modulus (E), shear modulus (G), and strain energy values (U). The enhanced aggregate stability observed could be attributed to these improvements in mechanical behavior. On the other hand, the hydraulic behavior showed various alterations in soil water retention capacity (WR), with BH displaying higher WR during swelling-drying cycles. Consequently, hydraulic conductivity experienced a marked reduction in bacterial alginate treatments when compared to algal alginate and control treatments. These transformations can be elucidated by the formation of a bridge-like structure between sand particles, as identified through bright field microscopy. This structure potentially alters the wettability properties of the treated soils, as evident in the differences in contact angle values. Our findings underscore that bacterial alginate hydrogel exerts a more pronounced impact on enhancing the mechanical and hydraulic properties of coarse quartz sand compared to traditional algal alginate. The application of soil conditioners serves as a valuable tool in mitigating soil erosion caused by factors such as climate change and tillage (e.g., wind erosion, heavy rainfall, and drought) [19,22,43]. One of the most used soil conditions in agricultural systems is the algal alginate [43]. However, the limitations of this hydrogel are related to the molecular properties, and consequently its gelation capacity, which can vary on seasonal climatic conditions, limiting the production of high-quality product for agricultural systems. Bacterial alginate has the advantage that it is produced under controlled conditions; however, few studies have focused on evaluating its application in agricultural soils [38,39].

To understand the mechanical behavior of soils treated with bacterial and algal alginate hydrogels, unconfined uniaxial compressive test (UUCT) and aggregate stability measurements were carried out. The stress-strain characteristics observed provide insights into the soil’s resistance and elasticity, which are vital mechanical properties for assessing soil stability [44]. Resistance is primarily influenced by internal factors such as particle size distribution, aggregation resulting from swelling-shrinkage behavior, the quantity and composition of organic matter, moisture content, pore density, and the type and quantity of adsorbed cations [2]. Previous studies demonstrated that the use of algal alginate hydrogels improves the resistance of different types of soils to compression stress [38,39,45,46,47,48]. The application of algal alginate (i.e., algal alginate) modified the mechanical properties of sand, where an increase in alginate concentration was directly correlated (r^2^ = 0.998) with a higher compressive strength [48]. Similar to prior studies, our observations revealed a direct correlation between the concentration of bacterial or algal alginate hydrogel and the enhancement of soil mechanical properties (Figure 1; Table 2). Notably, the treatment featuring the lowest concentration of bacterial alginate (BH1) exhibited a 38% higher yield stress and a 36% higher Young modulus compared to the treatment with the highest concentration of algal alginate (AH3) (Table 2). This implies that bacterial alginate may enhance the soil’s resistance to deformation. Soil aggregation, an essential soil physical property, requires consideration due to its significant influence on the hydro-mechanical characteristics of the soil. In this investigation, we assessed the aggregate stability of coarse quartz sand utilizing various concentrations of bacterial and algal alginate hydrogel. Based on the aggregate stability classification outlined by Le Bissonnais, the coarse quartz sand exhibited very low aggregate stability (MWD < 0.2 mm) [49]. Soils with these characteristics are more susceptible to erosion and runoff, and consequently have greater water permeability (i.e., high hydraulic conductivity and low water holding capacity). The application of bacterial and algal alginate hydrogels improved the stability of the aggregates, obtaining in both conditions an average aggregate stability of <0.9 mm, showing that alginate hydrogels can be useful to create aggregates in sand. The formation of a three-dimensional network between the quartz sand particles and the alginate hydrogels was observed (Figure 5). Soils treated with bacterial alginate hydrogel displayed more extensive bridging structures compared to soils treated with algal alginate hydrogel. The inherent lack of cohesion between sand particles in sandy soils is attributed to their limited presence of fine particles, such as clays, the absence of organic matter, and the rounded shape of sand grains [38,50,51]. Hence, the enhancements in mechanical properties observed in the BH treatments may be attributed to the increased interaction between the sand particles and the algal alginate hydrogel. These findings imply that bacterial alginate hydrogel can bolster the mechanical strength of coarse quartz sand in comparison to algal alginate hydrogel. Consequently, the utilization of bacterial alginate hydrogel could potentially play a pivotal role in agricultural systems contending with the challenges posed by climate change and agricultural soil tillage.

The mechanical effects of a hydrogel depend on its chemical and aggregation structure [52]. Alginates are composite polysaccharides comprising α-L-guluronate (G) and β-D-mannuronate (M) residues. The gelation properties of alginate depend on the structure of the alginate (M/G ratio, sequence, and length of the G block) and the molecular weight [26,35,53,54]. It is known that the molecular weight of alginate determines the rheological behavior of the material, in terms of its viscosity and the physical properties of the resulting gels [26,53]. The molecular weight of *A. vinelandii* alginate has a molecular weight of 453 ± 42 kDa [31]. Besides, alginates produced by bacteria are acetylated in O-2 and/O-3 positions in some mannuronate residues. Acetylated alginates show better interaction with water molecules than non-acetylated alginates [55,56]. Finally, the bacterial alginate hydrogel presented temperature and pH stability [39]. The structural differences in soils treated with bacterial alginate could offer an explanation for the superior mechanical properties observed. The soil structure is a primary determinant of hydraulic functions [2]. Sand, characterized by its larger pores and low organic matter content, naturally exhibits limited water retention capacity, resulting in enhanced hydraulic conductivity (i.e., higher flow and loss of water) [15,57,58,59]. The application of higher concentrations of both bacterial and algal alginate hydrogel in coarse quartz sand during tests for hydraulic conductivity and water retention capacity demonstrated a clear reduction in hydraulic conductivity and an increase in water retention capacity compared to the control treatment (Figure 3 and Figure 4). It’s noteworthy that bacterial and algal alginate hydrogels exhibited a hydrophilic nature, as indicated by contact angles measuring less than 25° (Figure 6). Additionally, a discernible relationship between hydrogel concentration and the formation of bridges between sand and hydrogel particles was observed under bright field microscopy (Figure 5). These findings align with previous research where soils treated with higher concentrations of algal alginate displayed a decrease in hydraulic conductivity, an augmentation in water retention capacity, and improvements in soil mechanical properties [45,60]. The hydrophilic nature of alginate hydrogels as well as the high-water retention capacity has been previously reported [61,62]. Barrientos et al. (2021) demonstrated that treating coarse quartz sand with 0.01% *w*/*w* bacterial alginate hydrogel led to a transition from the Forchheimer flow regime to a Darcy regime, which is in line with our hydraulic conductivity results. Additionally, soils treated with bacterial alginate hydrogel exhibited a high Ohnesorge number (Oh > 1), signifying the prevalence of viscous forces over inertia and surface tension forces, thereby enhancing the soil’s hydraulic properties [38,39]. A high Ohnesorge number allows hydrogel filaments to remain more stable due to increased viscosity, effectively retaining water within the filaments [63,64]. Given the variations in molecular weight between alginates, it is anticipated that bacterial alginate hydrogels will yield higher Ohnesorge values compared to algal alginate hydrogels. Furthermore, bacterial alginate hydrogels have the capacity to enhance water contact surface area through their coating, thus reducing pore space and augmenting the bonding strength between sand particles [38,45,46,47,65]. This capacity appears to be more pronounced when compared to algal alginate hydrogels.

## 4. Conclusions

In this study, we explored the impact of both bacterial and algal alginate hydrogels on the hydro-mechanical properties of coarse quartz sand. Our findings indicate that bacterial hydrogels applied to coarse quartz sand exert a more significant influence on mechanical properties, hydraulic conductivity, and water retention capacity. For instance, when 0.05% *w*/*w* of bacterial alginate hydrogel was applied to coarse quartz sand, a 23% increase in yield stress was achieved in comparison to the treatment using 0.15% *w*/*w* algal alginate hydrogel. Furthermore, at the same concentration of hydrogel, the BH treatments exhibited lower hydraulic conductivity and greater water retention capacity. Notably, bacterial alginate hydrogel boasts the advantage of maintaining stable molecular properties and, consequently, effectiveness as a soil conditioner when produced under controlled conditions. These results suggest that the utilization of bacterial alginate hydrogels in agricultural systems could be a promising candidate for soil conditioning, effectively mitigating the adverse consequences of erosion and intense tillage. Nonetheless, further research is warranted to comprehend how bacterial alginate hydrogels respond to different soil types and environmental factors, including pH, electrical conductivity, and temperature. Moreover, considering the structural modifications observed in soil treatments involving bacterial alginate hydrogel, future studies should concentrate on examining how these polymers interact with plants and the plant rhizosphere under abiotic stress conditions like water scarcity, salinity, and waterlogging.

## 5. Materials and Methods

### 5.1. Soil

In this study, coarse quartz sand (Migrin S.A) was selected as the soil medium for assessing the impact of alginate hydrogels due to its specific physical characteristics. These characteristics include a high susceptibility to compressive mechanical stresses, excellent permeability to water, and a lack of aggregate stability [15,64,66]. The sand is mainly made up of 55.46% particles with a particle size of 0.5 mm and 37.95% of 0.25 mm. The sand was classified as coarse grained [38].

### 5.2. Preparation of Ca-alginate Hydrogel

Two types of alginates were used in this study. Bacterial alginate was obtained from the batch cultures using *Azotobacter vinelandii* ATCC 9046 based on the same methodology reported [39]. This alginate has a molecular weight of 453 ± 42 kDa. In contrast, medium-viscosity algal alginate derived from Macrocystis pyrifera (AS) was sourced from Sigma-Aldrich Inc. In the hydrogel formation process, we employed CaCl_2_. When CaCl_2_ is diluted in water, it releases Ca^2+^ ions into the medium, making them available for the creation of the Ca-alginate porous complex, or hydrogel. Both the bacterial and algal alginate were dissolved in distilled water. Subsequently, a 0.5 M CaCl_2_ solution in filtered water was prepared and subjected to autoclaving at 121 °C and 0.1 MPa for 20 min. Finally, the alginate and CaCl_2_ solution were added to the quartz sand and mixed manually to ensure the homogeneity of the Ca-alginate hydrogel.

### 5.3. Sample Preparation

All the experiments were carried out in the Physicochemical & Environmental Plant Physiology laboratory at the Pontificia Universidad Católica de Valparaíso, Chile. The experimental design included three treatment groups: a control treatment (C), bacterial alginate hydrogel (BH), and algal alginate hydrogel (AH). Within each hydrogel treatment group, we employed three different levels, namely 0.05%, 0.10%, and 0.15% *w*/*w* of alginate relative to the coarse quartz sand. These levels were denoted as BH1, BH2, and BH3, respectively. These treatment groups remained consistent throughout all experiments conducted in this study. To create the hydrogel within the soil, the alginate solution and CaCl_2_ (0.5 M) were added separately in a 1:1 ratio, resulting in a final CaCl_2_ concentration of 0.25 M within the soil mixture. The volume of water applied was determined based on the sand’s saturation point, equivalent to its field capacity, which amounts to 150 mL of water for every 800 g of sand.

### 5.4. Determination of pH and Electrical Conductivity

The pH and electrical conductivity of the treatments were assessed using a pH/conductivity meter (HANNA HI2020-02, HANNA Instruments, Smithfield, RI, USA). A combination of 20 g of dry soil and distilled water in a 1:2 ratio was prepared. The mixture underwent vigorous stirring for 10 min and was subsequently left to settle for 1 h. The pH and electrical conductivity were then measured from the supernatant.

### 5.5. Unconfined Uniaxial Compression Test

A texture analyzer (Model Ta.XT plusC, Stable Micro Systems Ltd., Surrey, UK) equipped with a 100 mm diameter compression plate (TMS 100 mm diameter, p/100) was employed for both control and alginate hydrogel treatments. Prior to the compression test, the treatments were compacted within a custom-made mold, assuming a cylindrical shape measuring 2.6 cm in diameter × 2.8 cm in height, with a cylindrical area of 33.49 cm^2^ and a volume of 14.87 cm^3^. The texture analyzer software settings were configured as follows: pre-test = 0.1 mm/s, speed-test = 0.1 mm/s, post-test = 0.01 mm/s, force = 0.05 N. The trigger threshold was set based on force measurements, and compression force was recorded in Newtons (N) at a 2 mm deformation to accurately capture the elastic behavior of the treated soils. The yield strength, elastic modulus (E), shear modulus (G), and strain energy (U) were calculated using the same methodology as reported [39]. These parameters were determined based on the average of 5 repetitions per treatment.

### 5.6. Aggregate Stability Measurement

To assess aggregate stability in both the control and alginate hydrogel treatments, the mechanical breakdown method following a prewetting procedure was employed. The prewetting process serves to evaluate the wet mechanical cohesion of aggregates, independently of slaking [49]. Each experimental unit consisted of 250 g of soil, and five replications were carried out for each treatment. For a duration of two weeks, each experimental unit was subjected to 15 cycles of wetting (field capacity) and drying (permanent wilting point). The mechanical breakdown via shaking following the prewetting method was applied to each experimental unit. To execute this, 10 g of aggregates were gently immersed in 100 mL of 95% ethanol for 10 min. The ethanol was then drained, and the aggregates were transferred to an Erlenmeyer flask containing 200 mL of distilled water. The mixture was shaken 20 times and allowed to settle for 30 min. Excess distilled water was removed and transferred to a 50 μm sieve previously immersed in 95% ethanol for measurement of fragment size distribution. The samples were oven dried (BOV-C30T, BioBase Biodustry) at 60 °C for 24 h and then screened using seven sieves (2000, 1000, 500, 250, 125, and 53 μm). The mean weight diameter (MWD; g mm^−1^) was calculated as an index of aggregation using the next formula:

(1) $$MWD=\sum_{t=1}^{n}W_{i}\bar{X_{i}}$$

where *n* is the number of sieves, *W_i_* is the average diameter of each size fraction (mm), and *W_i_* is the proportion of the total sample weight occurring in the fraction *i* (g). 

### 5.7. Soil Water Retention Capacity

The capacity of the treatments to retain water in the soil was quantified in terms of water retention (WR). The gravimetric method was employed to ascertain WR, with the weight loss being measured at 12-h intervals until no significant difference was observed. Each experimental unit was comprised of 200 g of dry soil, and 37.5 mL of distilled water was added until saturation was achieved. Five replications were conducted for each treatment. WR was calculated as follows:

(2) $$WR=\frac{W_{t}-W}{W_{i}-W}\times 100\left[\%\right]$$

where W is the weight (g) of the sample without water, Wi is the weight of the sample after adding the water and Wt refers to the weight of the sample after specified time intervals. We assessed the re-swelling capacity of the alginate hydrogels. Each experimental unit underwent four cycles of wetting until saturation followed by four weeks of drying. In the last cycle, water retention (WR) was evaluated once more. Throughout these tests, temperature monitoring was conducted using a datalogger (Onset UA-002-64 HOBO, Pendant^®,^ Bourne, MA, USA).

### 5.8. Hydraulic Conductivity Test

The falling head permeability method was implemented following the ASTM D5084-16a protocol [67]. The experimental unit consisted of 700 g soil. The mixture of sand and water (control treatment) and sand and hydrogel (bacterial and algal alginate treatment) was left to rest for four days. After sample preparation, three consecutive falling head experiments (i.e., flushing events) were conducted on the different saturated soil media using the head permeability test set (HM-891, Gilson Company Inc., Lewis Center, OH, USA). Five replications were used for this experiment, and the average of the three flushing events was used to compute saturated hydraulic conductivity (*K*) using an adaptation of Darcy`s law equation [68]

(3) $$K=\frac{aL}{A(∆t)}*Ln\left(\frac{h_{0}}{h_{1}}\right)$$

where a is the cross-sectional area of standpipe (m), L is the length of specimen (m), A is the cross-sectional area of specimen (m), ∆t is time elapsed (s) and h_0_ and h_1_ are the initial and final water meniscus heights of the water column (m).

### 5.9. Contact Angle Measurements

We investigated the wettability characteristics of the various hydrogel treatments through contact angle measurements, employing a custom-made contact angle meter. Alginate hydrogels were applied to glass slides and subsequently dried in an oven at 35 °C for 24 h. Contact angles were determined using the sessile drop method, involving the placement of 1 µL drops of deionized water onto the sample surface at room temperature (25 °C). Contact angles were optically captured at the three-phase interface using a CMOS camera (Zelux 1.6 MP monochrome CS165MU1, THORLABS, Newton, NJ, USA) attached to a 12× zoom lens (Fine Focus and Coaxial Illumination Port MVL12X3Z, THORLABS, Newton, NJ, USA) for 30 s after the drop made contact with the sample surface. Angle values were calculated using ImageJ 1.53t software, and nine measurements were recorded for each treatment.

### 5.10. Bright Field Light Microscopy Imaging

Bright field light microscopy imaging was employed to examine the structure resulting from the interaction between coarse quartz sand and the various hydrogel treatments. The Ca-alginate hydrogel’s capacity to swell and absorb water facilitated the use of a blue ink-water solution (e.g., pen ink) to delineate the structure of the new matrix [13]. A 2% alginate solution containing 0.02% blue food-grade ink was prepared in advance. The mixture of alginate and quartz sand hydrogel was applied to glass slides. To observe the interaction of the hydrogel with the quartz sand, the samples were briefly dried in an oven at 70 °C to remove excess moisture. The calcium alginate hydrogel absorbed the % blue food-grade ink solution, allowing for its visualization under bright light microscopy. The samples were examined using a Leica DMIL LED inverted microscope (Leica Microsystems, Wetzlar, HE, Germany), and images were captured using a Leica MC170HD digital camera.

### 5.11. Statistical Analysis

ANOVA was conducted utilizing R version 2023.09.0+463, a statistical computing environment developed by R Core Team in 2023 and supported by R Foundation for Statistical Computing, Vienna, Austria. The CAR 3.1-2 software package [69] was employed for this analysis. To identify significant differences among treatments, Tukey’s honest significant difference test was utilized.

## Figures and Tables

**Table 1:** Effect of bacterial and algal alginate hydrogel concentration on the pH and electrical conductivity (EC) soil. Data are means ± SE (*n* = 5).

Treatment	pH	EC
W	6.77 ± 0.05	95.90 ± 2.68
C	6.85 ± 0.02	127.48 ± 2.68
AH1	6.46 ± 0.02	850.40 ± 5.82
AH2	6.44 ± 0.04	838.20 ± 28.67
AH3	6.54 ± 0.01	844.40 ± 12.96
BH1	7.50 ± 0.04	867.20 ± 6.37
BH2	7.53 ± 0.03	1336.80 ± 43.63
BH3	7.54 ± 0.03	1781.20 ± 53.13

**Table 2:** Summarized statistical data of the mechanical properties of control and alginate hydrogel treatments after the unconfined uniaxial compression test (UUCT). Data are means ± SE (*n* = 5).

Treatment	Yield Stress (MPa)	Yield Strain	E (MPa)	G (MPa)	U (Joules)
C	6.57 × 10^−4^ ± 4.42 × 10^−5^	0.071	9.21 × 10^−3^ ± 6.26 × 10^−4^	3.07 × 10^−3^ ± 2.09 × 10^−4^	3.49 × 10^−10^ ± 2.32 × 10^−11^
AH1	9.65 × 10^−4^ ± 7.91 × 10^−5^	0.071	1.35 × 10^−2^ ± 1.12 × 10^−3^	4.51 × 10^−3^ ± 3.72 × 10^−4^	5.11 × 10^−10^ ± 4.17 × 10^−11^
AH2	9.92 × 10^−4^ ± 1.02 × 10^−4^	0.071	1.39 × 10^−2^ ± 1.42 × 10^−3^	4.63 × 10^−3^ ± 4.73 × 10^−4^	5.26 × 10^−10^ ± 5.43 × 10^−11^
AH3	1.24 × 10^−3^ ± 9.92 × 10^−5^	0.071	1.75 × 10^−2^ ± 1.48 × 10^−3^	5.83 × 10^−3^ ± 4.94 × 10^−4^	6.56 × 10^−10^ ± 4.98 × 10^−11^
BH1	1.52 × 10^−3^ ± 3.68 × 10^−5^	0.071	2.13 × 10^−2^ ± 4.94 × 10^−4^	7.12 × 10^−3^ ± 1.65 × 10^−4^	8.08 × 10^−10^ ± 2.04 × 10^−11^
BH2	1.60 × 10^−3^ ± 9.18 × 10^−5^	0.071	2.25 × 10^−2^ ± 1.30 × 10^−3^	7.50 × 10^−3^ ± 4.33 × 10^−4^	8.47 × 10^−10^ ± 4.83 × 10^−11^
BH3	1.70 × 10^−3^ ± 8.49 × 10^−4^	0.071	2.41 × 10^−2^ ± 1.47 × 10^−3^	8.04 × 10^−3^ ± 4.90 × 10^−4^	8.93 × 10^−10^ ± 3.64 × 10^−11^
C	6.57 × 10^−4^ ± 4.42 × 10^−5^	0.071	9.21 × 10^−3^ ± 6.26 × 10^−4^	3.07 × 10^−3^ ± 2.09 × 10^−4^	3.49 × 10^−10^ ± 2.32 × 10^−11^
AH1	9.65 × 10^−4^ ± 7.91 × 10^−5^	0.071	1.35 × 10^−2^ ± 1.12 × 10^−3^	4.51 × 10^−3^ ± 3.72 × 10^−4^	5.11 × 10^−10^ ± 4.17 × 10^−11^
AH2	9.92 × 10^−4^ ± 1.02 × 10^−4^	0.071	1.39 × 10^−2^ ± 1.42 × 10^−3^	4.63 × 10^−3^ ± 4.73 × 10^−4^	5.26 × 10^−10^ ± 5.43 × 10^−11^
AH3	1.24 × 10^−3^ ± 9.92 × 10^−5^	0.071	1.75 × 10^−2^ ± 1.48 × 10^−3^	5.83 × 10^−3^ ± 4.94 × 10^−4^	6.56 × 10^−10^ ± 4.98 × 10^−11^

## Data Availability

All data and materials are available on request from the corresponding author. The data are not publicly available due to ongoing researches using a part of the data.

## Supplementary Materials

The following supporting information can be downloaded at: https://www.mdpi.com/article/10.3390/gels9120988/s1, Figure S1: Effect of alginate hydrogel concentration on soil pH and electrical conductivity. The (a) pH and (b) EC of the control (C), bacterial alginate (BH) and algal alginate hydrogel (AH) treatments are shown. Data are means ± SE (*n* = 5). Mean followed by different letters are significantly different by Tukey test. (*p* < 0.05); Figure S2: Mechanical properties of control and alginate hydrogel treatments after the unconfined uniaxial compression test (UUCT). Yield stress, Young`s modules (E), shear modulus (G) and strain energy (U) values of the treatments are presented into panels. Data are means ± SE (*n* = 5). Letters above the error bars indicate statistically significant differences between the treatments (*p* < 0.05); Figure S3: Water retention capacity of control and alginate hydrogel treatments during the (a) first and (b) fourth swelling–drying cycle. Data are means ± SE (*n* = 5). Letters above the error bars indicate statistically significant differences between the treatments (*p* < 0.05); Figure S4: Water retention capacity behavior of (a) AH1, (b) AH2, (c) AH3, (d) BH1, (e) BH2, (f) BH3 and (g) C treatment during the first and fourth swelling–drying cycle. Data are means ± SE (*n* = 5).
