# Supplementary material for: Enhancing Soil Resilience: Bacterial Alginate Hydrogel vs. Algal Alginate in Mitigating Agricultural Challenges
Source: Gels. 2023 Dec 17;9(12):988. doi: 10.3390/gels9120988 (PMC10743275; doi:10.3390/gels9120988)
Supplement: Supplementary file 1 [file gels-09-00988-s001.zip › gels-2748285-supplementary.pdf]

Supplementary Materials

Enhancing Soil Resilience: Bacterial Alginate Hydrogel vs. Algal Alginate in Mitigating Agricultural Challenges

Flavia Dorochesi, Cesar Barrientos-Sanhueza, Álvaro Díaz-Barrera and Italo F. Cuneo

The following Supporting Information is available for this article:

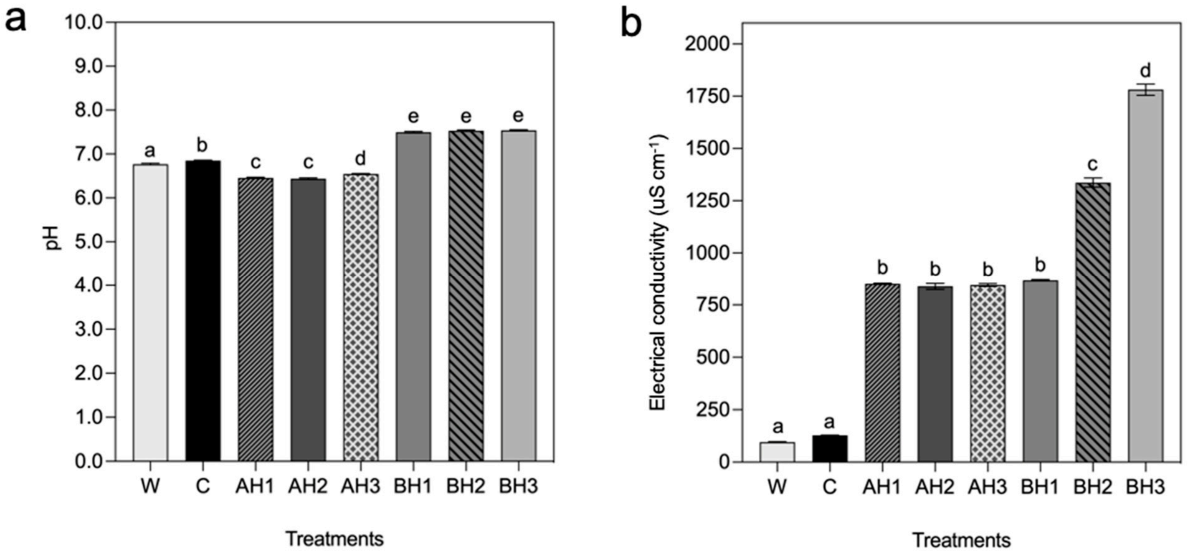

Figure S1. Effect of alginate hydrogel concentration on soil pH and electrical conductivity. The (a) pH and (b) EC of the control (C), bacterial alginate (BH) and algal alginate hydrogel (AH) treatments are shown. Data are means ± SE (n=5). Mean followed by different letters are significantly different by Tukey test. ($p<0.05$).

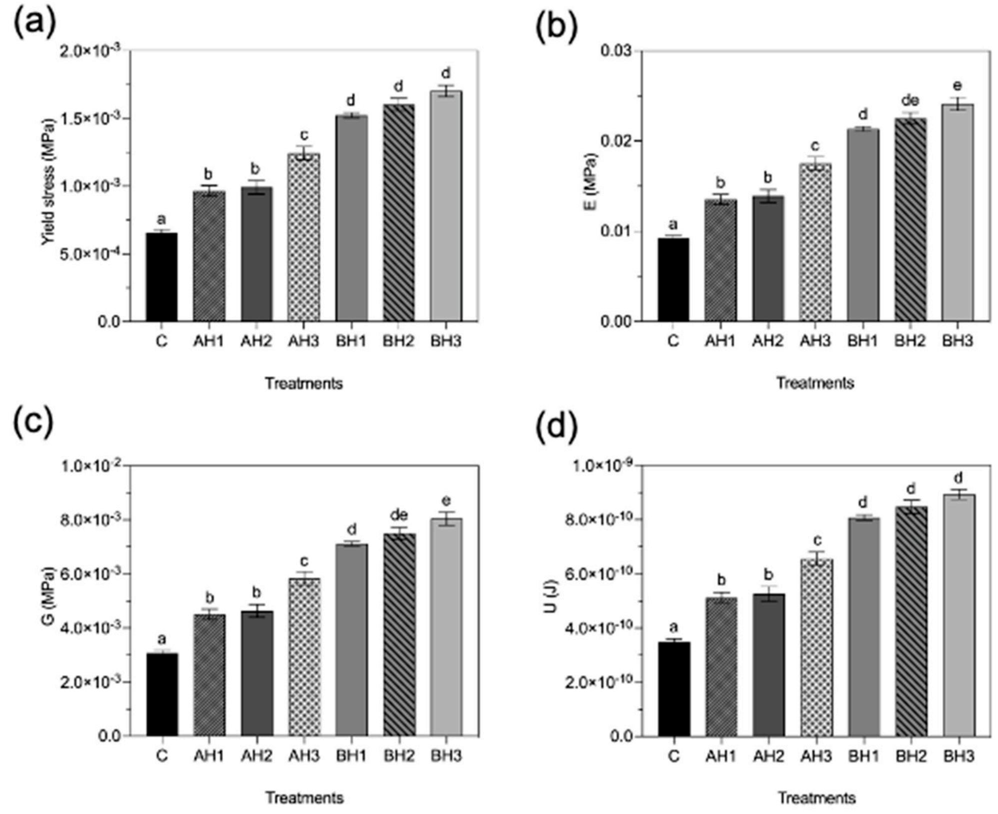

Figure S2. Mechanical properties of control and alginate hydrogel treatments after the unconfined uniaxial compression test (UUCT). Yield stress, Young`s modules (E), shear modulus (G) and strain energy (U) values of the treatments are presented into panels. Data are means ± SE (n=5). Letters above the error bars indicate statistically significant differences between the treatments ($p<0.05$).

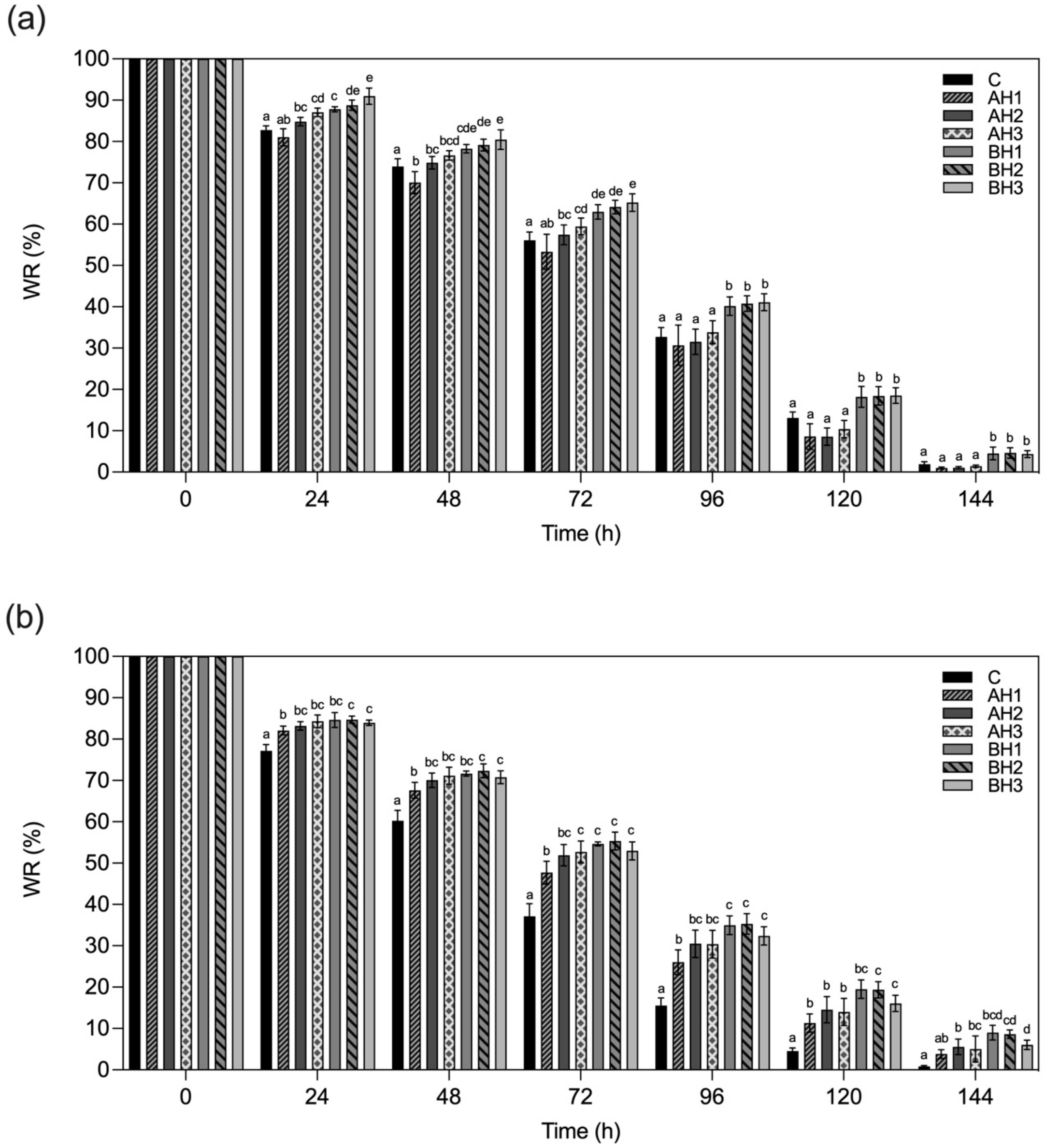

Figure S3. Water retention capacity of control and alginate hydrogel treatments during the (a) first and (b) fourth swelling–drying cycle. Data are means ± SE (n=5). Letters above the error bars indicate statistically significant differences between the treatments ($p<0.05$).

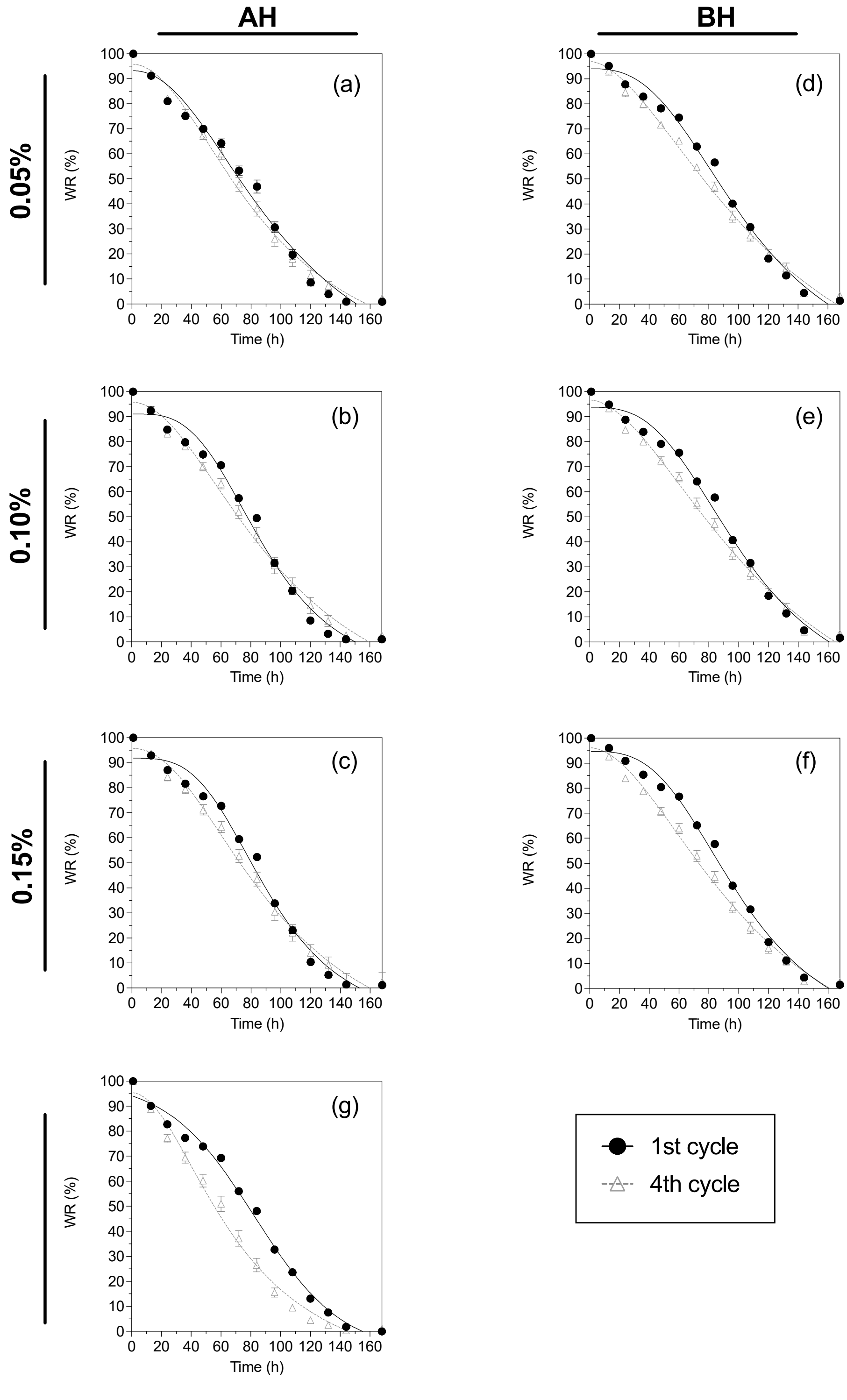

Figure S4. Water retention capacity behavior of (a) AH1, (b) AH2, (c) AH3, (d) BH1, (e) BH2, (f) BH3 and (g) C treatment during the first and fourth swelling–drying cycle. Data are means ± SE (n=5).
